# Research on the Contagion of Systemic Financial Risk Under the Impact of Climate Risks—From the Perspective of Complex Networks and Machine Learning

**DOI:** 10.3390/e28060711

**Published:** 2026-06-21

**Authors:** Xiao-Li Gong, Xiao-Han Sun, Sergey Aleksandrovich Philin

**Affiliations:** 1 School of Economics, Qingdao University, Qingdao 266061, China; 2 Laboratory of Complex Economic Systems and Digital Governance, Qingdao University, Qingdao 266061, China; 3 Higher School of Management, Plekhanov Russian University of Economics, 109992 Moscow, Russia

**Keywords:** climate risk, financial systemic risk, risk contagion, RF-AdaBoost model

## Abstract

To systematically examine the impact of climate risks on China’s financial system, this study employs the EGARCH-SGED model to precisely fit financial market volatility based on China’s Climate Change News Index. It then combines the LASSO-CoVaR method to measure tail risk spillover effects within China’s financial system under climate risk shocks, constructs a risk contagion network, and innovatively utilizes the RF-AdaBoost model to establish the risk early warning system. Findings reveal that climate risk is a key driver of dynamic correlation evolution within the financial system, with heterogeneous impacts across different markets. Physical climate risk events intensify short-term risk contagion while generating long-term effects; transition risks undergo a dynamic process, initially amplifying uncertainty before enhancing systemic stability over the long term. The RF-AdaBoost model outperforms traditional machine learning models in risk warning, demonstrating outstanding predictive accuracy and generalization capabilities, thereby providing effective intellectual support for climate risk prevention and financial stability management.

## 1. Introduction

As the global climate system continues to warm, the frequency and intensity of extreme weather events have risen significantly, posing a grave threat to human societies and economic systems [1]. The *Blue Book on Climate Change in China 2025* explicitly identifies China as a region highly sensitive to global climate change, where the impacts of such change are particularly pronounced. Climate risk is a complex systemic risk that arises directly or indirectly from climate change and, through multidimensional interactions among the physical environment, socioeconomic systems, and regulatory frameworks, poses potential threats to financial stability. Climate risk can be categorized into two core types: physical risk and transition risk. Among these, physical risks mainly refer to economic and financial losses directly caused by climate-related factors, characterized fundamentally by deep uncertainty, nonlinear transmission, and irreversibility. Examples include rising sea levels increasing the risk exposure of coastal assets and extreme weather events disrupting industrial supply chains. Transition risks, on the other hand, stem from potential risks accumulated in the economic and financial sectors due to factors such as policy adjustments, technological innovations, shifts in investor decisions, and changes in market demand preferences arising from the global transition to a low-carbon economy [2,3,4]. Systemic financial risk refers to the overall or partial damage to the functions of the financial system, which leads to the interruption or contraction of financial services or a sharp increase in financing costs. This risk spills outward through the interconnected chains of the system and has a serious negative impact on the real economy. Its essence stems from the negative externalities caused by the failure of financial institutions, financial markets or financial infrastructure. Its core characteristics are interconnectedness, contagion and nonlinear amplification [5,6,7]. Against the backdrop of intensified global climate change and the high interconnectedness of the financial system, the impact of climate factors on the financial system has gradually transformed from potential risks into real threats. The actual impact of “green swan” risks has far exceeded the general market expectations [8], and related issues have gradually become an important research topic in the field of finance [9,10,11]. With the increasing severity of the global climate crisis, climate change is no longer just an environmental issue, but an important factor affecting corporate business decisions, bank credit allocation and risk exposure of financial institutions. It may also further affect financial stability through the interconnected mechanisms within the financial system [12,13,14].

The evolution of climate risk into systemic financial risk is not instantaneous, but rather a dynamic process involving the transmission of shocks from the real economy to the financial system and their gradual accumulation and amplification. To understand how climate risk ultimately evolves into a systemic financial crisis, it is essential to first clarify the micro-transmission mechanisms that initially impact real businesses and local financial markets. Due to differences in risk attributes, the initial transmission paths of physical risks and transitional risks to the financial system exhibit heterogeneity. Physical climate risks affect the financial system through multiple channels, including the balance sheet channel, the credit supply channel, and the investor sentiment channel. Extreme weather events can damage physical assets, reduce operating revenues [15], depress financial asset values [16], and increase climate risk premiums on credit products such as mortgages [17]. At the same time, such events intensify claims pressure on the insurance sector [18], leading to a persistent deterioration in corporate balance sheets. These effects weaken corporate capital, profitability, and liquidity. As a result, credit quality declines, while delinquency and default risks increase [19], and debt financing capacity is reduced [20]. These risks then propagate to financial institutions, manifesting as tighter bank credit, deteriorating loan quality, and declining asset quality. Exposure to physical risks such as extreme weather events may also heighten investor awareness of climate risks and increase risk aversion [21]. This can trigger excessive sell-offs of securities issued by affected firms, depress their current market prices [22], and amplify asset price volatility, thereby exacerbating financial market instability. Transition risks, in turn, tend to intensify financial instability through asset price volatility, shifts in investor preferences, and changes in market expectations. Low-carbon transition policies impose asset depreciation pressures on carbon-intensive industries, reducing the value of related investment portfolios [23,24,25]. Such fluctuations can create mismatches between supply and demand, increase corporate default risk, and transmit losses to financial institutions [12,26]. As transition risks accumulate, market expectations adjust and investors increasingly favor environmentally sustainable assets. This shift further increases the likelihood of sharp declines in the asset prices of carbon-intensive firms [27,28]. It is evident that although physical risks and transformation risks differ in their sources of impact and transmission paths, they both first weaken the asset quality of the real economy and lead to damage to local financial institutions or severe fluctuations in individual financial markets.

However, the threat of climate risk to the financial system is not primarily manifested as localized losses suffered by individual financial institutions or specific industries, but rather as the potential for initial shocks to propagate and amplify within the financial system, ultimately evolving into systemic financial risk. The core mechanism of this evolution lies in the risk transmission and amplification effect formed by the complex interconnected networks within the financial system. The financial network plays a dual role as both a risk “transmitter” and an “amplifier” in this process. Climate risk shocks may continue to spread along the financial network and accumulate under the influence of risk feedback mechanisms, ultimately evolving into a systemic crisis affecting the entire financial system. Existing research indicates that initial losses from natural disasters or climate transition shocks may form a chain transmission through capital flows, debt relationships, and cross-shareholding relationships among financial institutions. It amplifies at each level within the financial network, thus evolving from localized risk into systemic financial risk [29,30,31,32]. Furthermore, the risk diffusion process is not a simple linear transmission. Its scope and amplification are also influenced by the structural characteristics of the financial network. In recent years, related research has further deepened the understanding of risk diffusion mechanisms from the perspectives of systemic correlation, behavioral feedback, and multi-layered networks. Studies on climate risk and financial stability have shown that climate risk may amplify local shocks by enhancing the risk correlation within the financial system [33]. While studies on complex networks and risk contagion have further revealed the important role of behavioral feedback, network structure, and cross-market linkage in the risk diffusion process [34,35]. Therefore, the evolution of climate risk into systemic financial risk is not the result of the simple accumulation of local market risks, but rather a process of continuous transmission and diffusion of risk shocks in the financial network. In this process, the correlation between different financial sub-markets provides channels for risk transmission, while behavioral feedback and market linkage mechanisms further strengthen the risk diffusion effect, causing local shocks to gradually evolve into systemic risks that affect the stability of the entire financial system.

To further reveal the formation and transmission process of systemic financial risks under the impact of climate risk, it is necessary to accurately quantify the risk interconnectedness of the financial system under extreme conditions. In traditional risk spillover research, Value-at-Risk (VaR) and Conditional Value-at-Risk (CoVaR) have been widely used to capture tail-risk dependence across markets [36]. However, against the backdrop of increasingly complex relationships within the financial system, the traditional CoVaR method is susceptible to the effects of increased variable dimensionality and multicollinearity when identifying a large number of potential risk relationships, thus reducing the accuracy of risk transmission relationship identification. To address this issue, existing research has incorporated the Least Absolute Shrinkage and Selection Operator (LASSO) into the VaR and CoVaR framework, identifying key tail risk relationships through variable screening and constructing a systemic risk network [37,38,39]. LASSO-CoVaR can effectively identify the direction of risk transmission and spillover intensity in high-dimensional financial systems. With the deepening research on risk contagion mechanisms, network analysis has been widely applied to systemic risk research, providing an important tool for characterizing complex financial relationships and risk transmission processes. Related studies have shown that network analysis can not only identify risk propagation paths and key risk nodes, but also reveal the impact of network structure characteristics on shock diffusion and systemic risk formation [40,41]. Although the above studies mainly focus on systemic risk relationships within financial institutions or financial markets and do not directly examine climate risk shocks, their research ideas on identifying high-dimensional tail risk relationships and constructing risk networks provide important methodological references for this paper’s analysis of risk contagion among multiple financial sub-markets under climate risk shocks. Building on this literature, this study employs the LASSO-CoVaR method to construct a tail-risk spillover network for China’s financial system under climate risk shocks. It aims to examine the interconnectedness of the financial system in this context.

Against the backdrop of increasingly prominent global climate risks, research into early warning systems for the contagion of systemic financial risks in China is of critical importance for enabling regulatory authorities to prevent systemic crises, financial institutions to strengthen risk resilience, and market participants to optimize strategic decision-making. Because systemic financial risk is characterized by high dimensionality, nonlinearity, and complex interactions, traditional linear prediction models often fail to adequately characterize the risk evolution process. In recent years, machine learning methods have been widely used in management, economics, finance, and other fields due to their ability to handle complex data structures and uncover nonlinear relationships [42,43,44,45]. Random Forest (RF) possesses strong capabilities in processing high-dimensional data, characterizing nonlinear relationships, and resisting overfitting, and is widely used in financial forecasting research. Compared to traditional methods such as logistic regression, linear regression, rolling window estimation, and shrinking estimation, Random Forest can more effectively identify nonlinear relationships and variable interaction effects in complex financial data. Related studies typically use metrics such as accuracy, area under the precision-recall curve, mean squared error, and hedging error to evaluate model performance, showing that Random Forest performs well in out-of-sample forecasting and prediction error control [46,47]. Therefore, Random Forest has good application potential in identifying and predicting the dynamic changes in complex financial risks. However, systemic financial risks often exhibit strong nonlinear, sudden, and heterogeneous characteristics under climate risk shocks, and a single Random Forest model may be insufficient to fully capture some complex risk patterns. Adaptive Boosting (AdaBoost), by iteratively adjusting sample weights, makes the model pay more attention to samples with larger prediction errors, thereby enhancing the model’s ability to identify complex risk characteristics. Furthermore, its algorithm design breaks through the dependence on specific weak learners, and its dynamic weight adjustment mechanism and real-valued hypothesis handling capability provide a theoretical basis and practical path for combining with various methods such as decision trees, neural networks, and support vector machines. Therefore, to improve the early warning capability of systemic financial risk contagion under climate risk shocks, this paper constructs an AdaBoost-Enhanced Random Forest (RF-AdaBoost). The Random Forest model is optimized using AdaBoost’s dynamic optimization mechanism, combining the robustness of Random Forest with the boosting capability of AdaBoost to further improve the accuracy and stability of regression predictions. Furthermore, this paper incorporates risk network characteristics, climate risk indicators, financial market variables, and macroeconomic state variables into the early warning model to test its effectiveness in early warning of systemic financial risks under climate risk shocks.

Although the aforementioned studies have laid a foundation for understanding climate-related financial risks, shortcomings persist regarding the measurement of systemic risk contagion and the provision of forward-looking early warnings. Therefore, this paper aims to answer the following two core questions: What are the characteristics of the risk correlations and transmission paths in China’s financial system under climate risk shocks, and how can effective early warning of systemic risk contagion be achieved? This paper uses the LASSO-CoVaR method to construct a dynamic tail risk spillover network of systemic risk in China’s financial system under climate risk shocks to study the correlation characteristics of China’s financial system under climate risk shocks, and further applies the RF-AdaBoost model for early warning of systemic financial risk under climate risk shocks. The main innovative contributions of this paper are as follows: First, this paper expands the research on climate finance risk at the level of data foundation and research objects. It introduces the daily China Climate Change News Index into the study of systemic risk in China’s financial system and incorporates the money market, capital market, commodity market, foreign exchange market, gold market, and real estate market into a unified analytical framework. This allows for a more dynamic depiction of the overall risk status of China’s financial system under climate risk shocks from a higher time frequency and a more systematic market coverage, providing empirical evidence from multiple financial sub-markets in China for climate finance risk research. Second, it supplements the empirical evidence on the interconnectedness of China’s financial system under climate risk shocks. This paper combines EGARCH-SGED volatility modeling, LASSO-CoVaR tail risk identification, and complex network analysis to construct a dynamic tail risk spillover network encompassing climate risk and multiple financial sub-markets. It identifies risk spillover relationships between different markets and examines the time-varying characteristics of risk correlations within the financial system under climate risk shocks, providing new empirical evidence for understanding the risk correlations and dynamic evolution characteristics of China’s financial system under climate risk shocks. Third, it combines climate risk contagion measurement with systemic risk early warning, supplementing research on climate finance risk early warning. Based on the construction of a dynamic tail risk spillover network under climate risk shocks, this paper incorporates risk network characteristics, climate risk indicators, financial market variables, and macroeconomic state variables into an improved RF-AdaBoost model to predict the dynamic changes in systemic financial risk, providing empirical evidence for the early identification and risk warning of systemic financial risk under climate risk shocks.

**Figure 1:**
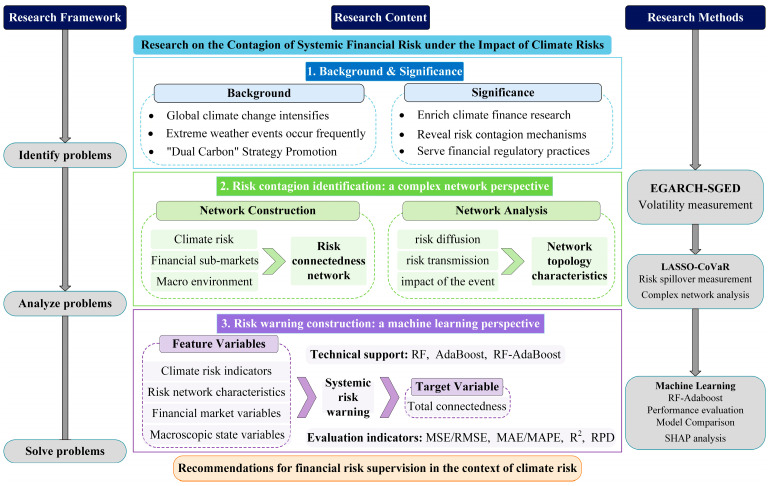
Research Framework Diagram.

To more intuitively illustrate the research ideas and technical approach of this paper, Figure 1 shows the overall research framework.

## 2. Theoretical Model

### 2.1. Measurement of Systemic Risk

Under the impact of extreme events, the spillover effects of tail risk among the various sub-markets within the financial system are significantly amplified. Returns data frequently deviates from a standard normal distribution, exhibiting typical characteristics such as leptokurtosis, heavy tails, and skewness, while also being accompanied by volatility clustering and heteroskedasticity. In this situation, directly using returns is insufficient to fully reflect changes in risk levels during extreme risk events, while volatility can more accurately characterize the accumulation and release of risk. Tail risk often does not originate from a single market fluctuation but gradually accumulates when the market is in a state of sustained high volatility. GARCH (Generalized Autoregressive Conditional Heteroskedasticity)-type models can utilize historical volatility information to characterize this risk persistence characteristic, thus providing an effective basis for tail risk measurement. However, in real financial markets, the impact of negative and positive shocks on volatility is often asymmetrical. Compared to positive news of equal magnitude, negative news is usually more likely to trigger market panic and risk contagion, i.e., there is a significant leverage effect. The EGARCH (Exponential GARCH) model, by introducing an asymmetric term to characterize the differentiated impact of positive and negative shocks on volatility, and by using the logarithm of conditional variance for modeling, does not require the imposition of non-negativity constraints on parameters, thus exhibiting greater flexibility and applicability. Therefore, in order to accurately characterize the peak, heavy tail, and skewed characteristics of tail risk in China’s financial system under climate risk shocks. To provide a reliable risk measurement basis for subsequent research on the contagion of systemic risk in China’s financial system under climate risk shocks, this paper selects the EGARCH model to fit the volatility of the return series of each financial sub-market, assuming that the innovation term *z_t_* follows a skewed generalized error distribution (SGED). The model is specified in Equation (1).

(1) $$\begin{array}{l} y_{t}=\mu_{t}+\varepsilon_{t},\varepsilon_{t}=\sqrt{h_{t}}z_{t}\\ \ln h_{t}^{2}=\alpha +\sum_{i=1}^{m}p_{i}\ln h_{t-i}^{2}+\sum_{j=1}^{n}q_{j}\left|\frac{\varepsilon_{t-j}}{h_{t-j}}\right|+\gamma_{j}\frac{\varepsilon_{t-j}}{h_{t-j}} \end{array}$$

where $\ln h_{t}^{2}$ is the logarithm of the conditional variance, $\varepsilon_{t}$ is the residual from the ARIMA (Autoregressive Integrated Moving Average) model, and $\varepsilon_{t}/h_{t}$ is the standardized residual of the EGARCH model. The EGARCH model captures the asymmetry in returns volatility by introducing the leverage effect coefficient γ, and relaxes the non-negativity constraints on the parameters p and q, thereby improving model applicability and flexibility. We assume that the innovation term *z_t_* follows a skewed generalized error distribution, and its probability density function is given in Equation (2).

(2) $$f(x\left|\mu ,h,v,\lambda \right.)=\frac{v}{2\theta h}\Gamma {\left(\frac{1}{v}\right)}^{-1}\exp(-\frac{1}{1+sign(x-\mu +\delta h)\lambda^{v}\theta^{v}h^{v}}\left|x-\mu +\delta h\right|)$$

where λ denotes the skewness parameter, v represents the tail-thickness parameter of the returns distribution, $\theta =\Gamma {\left(1/v\right)}^{0.5}\Gamma {\left(3/v\right)}^{-0.5}S{\left(\lambda \right)}^{-1}$, $\delta =2\lambda AS{\left(\lambda \right)}^{-1}$, $A=\Gamma (2/v)\Gamma {\left(1/v\right)}^{-0.5}\Gamma {\left(3/v\right)}^{-0.5}$, $S=\sqrt{1+3\lambda^{2}-4A\lambda^{2}}$, and Γ(.) denotes the Gamma function.

The essence of systemic risk lies in the continuous propagation and diffusion of risk shocks through the internal relationships within the financial system, impacting overall financial stability. The interconnected structure of the financial system is a crucial carrier for risk transmission and the formation of systemic risk [48,49,50]. Therefore, to identify the propagation paths, key risk sources, and risk-bearing entities of tail risks within the financial system under climate risk shocks from a network perspective, this paper constructs a systemic risk spillover network within China’s financial system under climate risk shocks following Hautsch et al. [37]. The systemic risk spillover network referred to in this paper is a weighted directed network constructed based on tail risk spillover relationships, used to characterize the propagation structure of internal risks within the financial system under climate risk shocks. Climate risk and various financial sub-markets constitute network nodes, and the edges between nodes and their weights are determined by the marginal risk spillover effect estimated by the LASSO-CoVaR model, reflecting the direction and intensity of risk transmission. In this network model, the marginal effects estimated using the LASSO-CoVaR method are employed as edge weights. Fundamentally, this approach follows a two-stage quantile regression procedure. First, VaR is estimated via quantile regression. Second, the LASSO algorithm is employed to identify tail-risk drivers most closely associated with systemic linkages, from which marginal effects *β* and CoVaR are estimated. Finally, based on the estimated marginal effects, a systemic financial risk contagion network is constructed, and its connectedness and topological characteristics are analyzed over time.

VaR is a widely used measure of risk. This paper employs quantile regression to estimate the VaR of the Climate Risk Index and various financial sub-markets. Let $R_{t}^{i}$ denote the volatility of the climate risk index or the financial markets at time t, then its VaR at the quantile level q is defined as follows.

(3) $$\;\mathrm{Pr}(R_{t}^{i}\leq VaR_{q,t}^{i})=q$$

To further assess the risk spillover effects of a single market on other markets as well as the overall financial system, this paper adopts the CoVaR measure proposed by Adrian et al. [36]. Conditional on a set of tail-risk drivers, CoVaR is defined as the value at risk of variable *j* conditional on variable *i* being in distress. Specifically, it measures the risk of variable *j* when the loss of variable *i* equals its $VaR_{q,t}^{i}$.

(4) $$Pr\left(R_{t}^{j|X_{t}^{\left(i\right)}}\leq CoVaR_{q,t}^{j|X_{t}^{\left(i\right)}}\right)=q$$

where $X_{t}^{\left(i\right)}$ denotes the information set, including $R_{t}^{j}=VaR_{q,t}^{i}$ and macroscopic state variables $M_{t-1}$. CoVaR is estimated using a two-stage quantile regression approach, which enables the modeling of conditional quantiles at a specified probability level. Unlike traditional mean regression, which focuses on the conditional mean, quantile regression directly characterizes the changes in the conditional distribution at different locations, making it more suitable for estimating tail risk indicators such as VaR and CoVaR. A key premise of this paper’s use of quantile regression is the heterogeneity of the impact of different risk drivers on different quantiles of financial market returns; that is, the same shock may have different effects under normal market conditions and extreme risk conditions. Since climate risk and market shocks often exhibit a stronger amplification effect in the tail phase, quantile regression can more effectively identify risk transmission relationships under extreme risk conditions.

Based on quantile regression, $VaR_{q,t}^{i}$ and $CoVaR_{q,t}^{i}$ can be obtained as follows.

(5) $$VaR_{q,t}^{i}={\hat{\beta }}_{q,0}^{i}+{\hat{\beta }}_{q,M}^{i}M_{t-1}$$

(6) $$CoVaR_{q,t}^{j|i}={\hat{\beta }}_{q,0}^{j|i}+{\hat{\beta }}_{q,M}^{j|i}M_{t-1}+{\hat{\beta }}_{q,VaR}^{j|i}VaR_{q,t}^{i}$$

Given data availability, the target market’s own lagged volatility, lagged macroeconomic state variables, and other relevant market variables are selected as potential drivers of tail risk. Specifically, the CoVaR of variable j at time t=1,…,T is defined as a linear function of its specific tail risk drivers $X_{t}^{\left(j\right)}$.

(7) $$CoVaR_{q,t}^{j|X_{t}^{\left(j\right)}}={\hat{\beta }}_{q,0}^{j|X_{t}^{\left(j\right)}}+{\hat{\beta }}_{q,M}^{j|X_{t}^{\left(j\right)}}M_{t-1}+{\hat{\beta }}_{q,VaR}^{j|X_{t}^{\left(j\right)}}VaR_{q,t}^{-j}$$

where $X_{t}^{\left(j\right)}\equiv \{1,M_{t-1},VaR_{q,t}^{-j}\}$, and ${\hat{\beta }}_{q}^{j|X_{t}^{\left(j\right)}}\equiv \{{\hat{\beta }}_{q,0}^{j|X_{t}^{\left(j\right)}},{\hat{\beta }}_{q,M}^{j|X_{t}^{\left(j\right)}},{\hat{\beta }}_{q,VaR}^{j|X_{t}^{\left(j\right)}}\}$ denote the vector of regression coefficients. $VaR_{q,t}^{-j}\equiv \{VaR_{q,t}^{1},\ldots ,VaR_{q,t}^{j-1},VaR_{q,t}^{j+1},\ldots ,VaR_{q,t}^{N}\}$ represents the set of VaR values for all variables excluding variable j.

However, not all variables’ VaR affect the CoVaR of variable j. Moreover, the tail-risk drivers $X_{t}^{\left(j\right)}$ specific to variable j cannot be directly identified, thereby raising the issue of variable selection. Although traditional variable screening methods (such as information-criterion-based selection) can be employed to identify the optimal subset of variables from a given set, they are procedurally complex to mitigate the issue of high multicollinearity among covariates. Consequently, they are unsuitable for multivariate selection and high-dimensional network modeling. To address this issue, this paper adopts the LASSO method, a data-driven statistical shrinkage technique, for covariate selection. This method is widely used in high-dimensional mean regression, and Belloni et al. [51] extended it to the quantile regression framework. The method facilitates the attainment of sparse solutions by applying an L_1_-norm regularization penalty term to the objective function of quantile regression, as follows:

(8) $${\hat{\beta }}_{q}^{j|X_{t}}=\arg\min\frac{1}{T}\sum_{t=1}^{T}\left[p_{q}\left(R_{t}^{i}-X_{t}^{\prime }\beta_{q}^{j|X_{t}}\right)+\lambda^{j}\frac{\sqrt{q\left(1-q\right)}}{T}\sum_{k=1}^{K}{\hat{\sigma }}_{k}|\beta_{q,k}^{j|X_{t}}\right]$$

where $X_{t}=\left\{X_{t,k}|k=1,\ldots ,K\right\}$ is a set of potentially relevant regressors, $\lambda^{j}$ is a fixed individual penalty parameter, and ${\hat{\sigma }}_{k}=1/T\sum_{t=1}^{T}{\left(X_{t,k}\right)}^{2}$ captures the variation in potential risk factors. The loss function is defined as $\rho_{q}\left(u\right)=u\left(q-I\left(u<0\right)\right)$, where $I\left(.\right)$ is an indicator function that equals 1 if u<0 and 0 otherwise, and K denotes the number of parameters. The value of $\lambda^{j}$ is determined in a data-driven manner. Its magnitude significantly influences the quality of variable selection, and when $\lambda^{j}=0$, LASSO regression reduces to standard quantile regression. Following Belloni et al. [51], a data-driven computational approach is employed for the selection of risk drivers of each variable—specifically, the magnitude of the penalty factor is determined entirely based on the model fit to the data.

### 2.2. Tail Risk Spillover Network

Based on the marginal effects estimated in the preceding steps, the time-varying CoVaR for each variable can be calculated, thereby enabling the construction of a dynamic tail-risk spillover network for China’s financial systemic risk under the impact of climate risks. To dynamically characterize the network structure, a sliding window approach is employed to partition the full sample into *W* windows, with a window size of *S* = 240 (corresponding to approximately one year’s worth of daily trading data), yielding the dynamic CoVaR values for each respective variable. Based on the LASSO-CoVaR framework, the relevant regressors can be obtained.

(9) $$CoVaR_{q|t}^{j|{\tilde{X}}_{t}^{\left(j\right)}}={\tilde{X}}_{t}^{{\left(j\right)}^{\prime }}{\hat{\beta }}_{q}^{j|{\tilde{X}}_{t}^{\left(j\right)}}$$

where ${\tilde{X}}_{t}^{\left(j\right)}\equiv \left\{1,M_{t-1},VaR_{q,t}^{-j|R}\right\}$, and $VaR_{q,t}^{-j|R}$ denotes a subset of $VaR_{q,t}^{-j}$. The corresponding coefficient vector is given by ${\hat{\beta }}_{q,VaR_{q,t}^{-j|R}}^{j|{\tilde{X}}_{t}^{\left(j\right)}}$.

To construct a tail risk spillover network, we first introduce the fundamental concepts of network theory. The network is composed of nodes and edges. If the edges are non-directional, it is an undirected network. If the edges possess directionality, it is a directed network. Furthermore, if each edge is assigned a specific weight, it constitutes a weighted network. In this study, we select climate risk and ten financial sub-markets to serve as the eleven nodes of the network. The marginal effect value β between variables i and j constitutes the edge connecting nodes i and j, reflecting the intensity of the risk shock that variable i exerts upon variable j, or conversely, the intensity of the risk shock that variable i receives from variable j. Consequently, the constructed tail risk spillover network is a weighted directed network.

Let the tail risk spillover network *G* consist of a set of nodes $V=\left\{v_{1},v_{2},\ldots ,v_{N}\right\}$ and a set of directed edges *E*: G=(V,E). If $VaR_{q,t}^{i}$ is selected as a risk driver by the LASSO algorithm, it indicates that variable j is influenced by variable i, implying the existence of a tail risk spillover effect from variable i to variable j. Specifically, ${\hat{\beta }}_{q,i}^{j|X_{t}^{\left(j\right)}}$ quantifies the degree to which variable j is influenced by variable i within the network system, thereby reflecting the impact of $VaR^{i}$ on $CoVaR^{j|i}$. Conversely, if $VaR_{q,t}^{i}$ is not selected as a risk driver, then no risk spillover effect exists from variable i to variable j. By iteratively applying this procedure, the tail risk spillover network *G* of China’s systemic financial risk under climate risk shocks can ultimately be constructed. The elements of its weighted adjacency matrix *A* are defined as follows:

(10) $$A_{i,j}=\left\{\begin{array}{l} |{\hat{\beta }}_{q,VaR_{q,t}^{i}}^{j|X_{t}^{\left(j\right)}}|,if\;VaR_{q,t}^{i}\;is\;selected,i.e.,\langle v_{i},v_{j}\rangle \in E\\ 0,if\;VaR_{q,t}^{i}\;is\;not\;selected,i.e.,\langle v_{i},v_{j}\rangle \notin E \end{array}\right.$$

where ${\hat{\beta }}_{q,VaR_{q,t}^{i}}^{j|X_{t}^{\left(j\right)}}$ denotes the *i*-th element of the vector ${\hat{\beta }}_{q,VaR_{q,t}^{-j|R}}^{j|X_{t}^{\left(j\right)}}$.

### 2.3. Connectedness Network Topology Feature Metrics

To comprehensively characterize the structural features of the risk transmission network in the financial system under the impact of climate risk, this paper constructs a network topology indicator system from two levels: direct risk association and indirect risk transmission. Direct risk association reflects the risk transmission between markets through network edges, measured by edge weights and their derivative indicators. Indirect risk transmission reflects the process of risk spreading through one or more intermediate nodes in the network, characterized by network centrality indicators. Based on this, this paper selects five indicators—Total Connectedness (TC), Risk In-strength (RIS), Risk Out-strength (ROS), Betweenness Centrality (BC), and PageRank Centrality (PR)—at both the system-wide and node-specific levels to analyze the characteristics of risk transmission.

(1) Total Connectedness (TC): Measured at the systemic level, this metric quantifies the aggregate intensity of tail risk spillovers across the entire network, thereby serving to gauge the overall connectedness of the system.

(11) $$TC=\sum_{j=1}^{N}\sum_{i=1,i\neq j}^{N}\left|{\hat{\beta }}_{q,VaR_{q,t}^{i}}^{j\left|{\tilde{X}}_{t}^{\left(j\right)}\right.}\right|$$

(2) Risk In-strength (RIS): Measured at the market level, this metric quantifies the intensity of tail risk spillovers flowing from all other markets into a specific market j, thereby reflecting the level of tail risk inflow to which market j is exposed.

(12) $$RIS_{j}=\sum_{i=1,i\neq j}^{N}\left|{\hat{\beta }}_{q,VaR_{q,t}^{i}}^{j\left|{\tilde{X}}_{t}^{\left(j\right)}\right.}\right|$$

(3) Risk Out-strength (ROS): Measured at the market level, this metric quantifies the intensity of tail risk spillovers flowing from a specific market j to all other markets, thereby quantifying the level of tail risk outflow originating from market j.

(13) $$ROS_{j}=\sum_{i=1,i\neq j}^{N}\left|{\hat{\beta }}_{q,VaR_{q,t}^{j}}^{i\left|{\tilde{X}}_{t}^{\left(i\right)}\right.}\right|$$

(4) Betweenness Centrality (BC) measures the degree to which a node is located in the risk propagation path of other nodes at the market level, and is used to identify key bridging nodes in the cross-market risk transmission process. Unlike RIS and ROS, which mainly characterize direct risk inputs and outputs, betweenness centrality can reflect the indirect risk spillover effects formed by risk transmission through intermediate nodes. It is defined as:

(14) $$BC_{i}=\sum_{s\neq i\neq t}\frac{\sigma_{st}(i)}{\sigma_{st}}$$

where $\sigma_{st}$ represents the number of shortest paths between node *s* and node *t*, and $\sigma_{st}(i)$ represents the number of these shortest paths that pass through node *i*. The larger the $BC_{i}$ value of a node, the more frequently that node is located on the risk propagation path between other markets, and the stronger its mediating role in the cross-market risk diffusion process.

(5) PageRank Centrality (PR) measures the systematic importance of a node in the entire risk contagion network. PageRank considers not only the direct risk associations of nodes but also the multi-level transmission effects of risk through network paths, thus reflecting the combined influence of direct and indirect risk spillovers and the systematic importance of nodes. It is defined as follows:

(15) $$PR_{i}=\frac{1-d}{N}+d\sum_{j\in M_{i}}\frac{PR_{j}}{L_{j}}$$

where *N* represents the total number of nodes in the network; *d* is the damping coefficient, usually taken as 0.85; *M_i_* represents the set of nodes pointing to node *i*; *PR_j_* represents the PageRank value of node *j*; and *L_j_* represents the number of outgoing chains or out-degree of node *j*. In the formula, (1 − *d*)/*N* represents the probability term for a node to randomly acquire basic influence, and the latter term represents the risk influence contribution that node *i* receives from its neighboring node *j*. The PageRank value is calculated iteratively, that is, each node is first assigned the same initial value, and then continuously updated according to the network connection relationship until the result converges. If the *PR_i_* value of a certain node is larger, it indicates that the node has a higher systemic importance in the entire risk contagion network, and its risk influence can be further diffused and accumulated in the financial system through direct association and multiple indirect paths.

### 2.4. Machine Learning Based on Regression Problems

#### 2.4.1. Random Forest Algorithm (RF) for Regression Problems

Random Forest is a quintessential ensemble learning model. Its core principle involves combining the predictive outputs of multiple decision trees (base models). In regression tasks, this method derives the final output by averaging the predictions of these individual trees. By introducing randomness in both sample selection and feature selection, Random Forest reduces the correlation among the base models, thereby enhancing the model’s generalization ability and robustness. The specific steps are as follows:

1. Bootstrap Sampling: Multiple samples are repeatedly and randomly drawn from the original dataset using sampling with replacement to generate a set of distinct sub-datasets.
2. Decision Tree Construction: For each sub-dataset generated during the Bootstrap sampling rounds, a regression tree is constructed using a decision tree algorithm. At each node split, a random subset of features is selected to serve as the feature set for that specific decision tree. The decision tree is built based on this training subset and feature set until a predetermined number of leaf nodes is reached or until further splitting becomes impossible. These steps are repeated to construct multiple decision trees. For a new input sample, it is fed into each of these decision trees to obtain multiple distinct prediction results.
3. Ensemble Prediction: Once all trees have been trained, for a given sample requiring prediction, each decision tree is used individually to generate a prediction; the results from all trees are then averaged to yield the final predicted value. The algorithmic formulation is based on the decision tree regression model, wherein the prediction function for each individual decision tree can be expressed in the following.
  (16) $$f_{n}=\sum_{j=1}^{J_{n}}c_{nj}I(x\in R_{nj})$$
  where n denotes the index of the decision tree, x represents the input sample, and $J_{n}$ denotes the number of leaf nodes in the *n*-th decision tree. $c_{nj}$ represents the predicted value of the j-th leaf node in the *n*-th decision tree, and $R_{nj}$ denotes the set of samples falling into the *j*-th leaf node of the *n*-th decision tree. $I\left(.\right)$ is the indicator function, defined such that $I(x\in R_{nj})=1$ *if* $x\in R_{nj}$, and $I(x\in R_{nj})=0$ otherwise.

The prediction function for multiple decision trees can be expressed as: $f(x)=\frac{1}{K}\sum_{k=1}^{K}f_{k}(x)$, where *K* denotes the number of decision trees.

#### 2.4.2. Adaptive Boosting Algorithm (AdaBoost) for Regression Problems

The Adaptive Boosting (AdaBoost) algorithm is a classic Boosting method within the field of ensemble learning. Its core logic adheres to the “additive model + forward stagewise optimization” paradigm: it sequentially trains a series of weak learners through iterative steps, dynamically adjusting both the sample weights and the weights assigned to each learner based on their respective performance, and ultimately combines them via weighted fusion to form a strong learner. In the context of regression tasks, this algorithm progressively refines the model’s ability to fit complex data distributions by focusing specifically on “hard samples”—those associated with large prediction errors—thereby effectively enhancing the model’s predictive accuracy and generalization capability regarding unseen data. Consider the following dataset, which consists of m samples: $D=\left\{\left(x_{1},y_{1}),(x_{2},y_{2}),\ldots ,(x_{m},y_{m}\right)\right\}$, where $x_{i}\in {\mathbb{R}}^{d}$ (each sample comprising a specific number of features) and $y_{i}$ denotes the target value corresponding to sample $x_{i}$. The specific steps are as follows:

1. Initialization of weights. Let the initial sample distribution be denoted by $Dist_{1}$ , where each sample $x_{i}$ is assigned an equal weight of $\frac{1}{m}$, that is, $Dist_{1}(x_{i})=\frac{1}{m}$. The distribution $Dist_{1}$ is used to train the first weak classifier $h_{1}$, while the distribution $Dist_{t}$ is used to train the *t*-th weak classifier $h_{t}$.
2. Iterate for T rounds. Let *t* denote the index of the weak classifier in each iteration, where $t\in \left\{1,2,3,\ldots ,T\right\}$. Each iteration proceeds according to the following steps:
  - Based on the current sample distribution $Dist_{t}(x)$, train a weak classifier $h_{t}$ on dataset *D*;
  - Calculate the maximum error $E_{t}$ of the classifier $h_{t}$ on the training set *D*, defined as follows:
    (17) $$E_{t}=\max\left|y_{i}-h_{t}(x_{i})\right|,i=1,2,\ldots ,m$$
    where $h_{t}(x_{i})$ denotes the prediction result of the weak classifier $h_{t}$ for sample $x_{i}$, and $y_{i}$ denotes the target value of the sample $x_{i}$.
  - Based on the maximum error *E_t_* obtained for $h_{t}$, calculate the relative error of $h_{t}$ for each sample; here, the squared error is used as an example:
    (18) $$e_{ti}=\frac{{\left(y_{i}-h_{t}(x_{i})\right)}^{2}}{E_{t}^{2}},i=1,2,\ldots ,m$$
  - Based on the sample relative error $e_{ti}$ obtained in the previous step, calculate the error rate of the current weak classifier $h_{t}$ (i.e., the sum of the products of the weights and errors of all samples in the dataset):
    (19) $$e_{t}=\sum_{i=1}^{m}Dist_{t}(x_{i})e_{ti}$$
  - Update the weights of the current weak classifier $h_{t}$, using the following formula:
    (20) $$w_{t}=\frac{e_{t}}{1-e_{t}}$$
  - Update the weight distribution of the dataset samples; for a sample $x_{i}$, the formula for updating the weight is:
    (21) $$Dist_{t+1}(x_{i})=\frac{Dist_{t}(x_{i})}{Z_{t}}w_{t}^{1-e_{ti}}$$
    where $Z_{t}$ is the normalization factor, calculated as follows:
    (22) $$Z_{t}=\sum_{i=1}^{m}Dist_{t}(x_{i})w_{t}^{1-e_{ti}}$$
  - Set t=t+1, and return to Step a within the loop body.
3. The *T*-th iteration concludes, yielding the final strong regressor as follows:
  (23) $$H(x)=\sum_{i=1}^{m}\ln(\frac{1}{w_{t}})f(x)=[\sum_{i=1}^{m}\ln(\frac{1}{w_{t}})]f(x)$$
  where f(x) is the median of all $w_{t}h_{t}(x)(t=1,2,\ldots ,T)$—that is, the median of the weighted outputs of all weak learners.

#### 2.4.3. AdaBoost-Enhanced Random Forest Algorithm for Regression Problems (RF-AdaBoost)

This paper integrates the Random Forest algorithm with the AdaBoost framework, utilizing Random Forests as base learners to perform ensemble learning in conjunction with the AdaBoost algorithm, thereby proposing an improved Random Forest model termed RF-AdaBoost. By combining the stochastic feature selection and bootstrap sampling capabilities of Random Forests with the ensemble advantages of AdaBoost, the RF-AdaBoost model effectively enhances the accuracy and robustness of data regression predictions. Its core concept is as follows: first, multiple Random Forests are trained to serve as base learners. Subsequently, the AdaBoost algorithm is employed to form a weighted combination of these Random Forests, ultimately yielding an ensemble model with superior predictive accuracy. The specific steps are outlined below:

1. Initialize parameters and sample weights. Given a dataset with m samples, each with *d* features and one target regression value, the initial weights of all samples are set equally. Determine the number of AdaBoost iterations, *K* (i.e., the number of base learners in the Random Forest); each Random Forest consists of *T* decision trees, and the depth of each individual decision tree is constrained by a preset value (ensuring that the Random Forest acts as a weak learner and preventing excessive depth or complexity that may lead to overfitting or numerical instability).
2. Iteratively train the Random Forest base learner. Train a Random Forest model based on the current sample weight distribution. Calculate the prediction error of the current Random Forest on the training set, including the maximum error and the relative error for each sample. Calculate the weighted error rate of the Random Forest based on the current sample weights. Assign a weight to the current Random Forest according to the weighted error rate. Increase the weight for samples with large prediction errors and decrease the weight for samples with small errors, and ensure that the weight sums to 1 through normalization, so that the next iteration focuses more on hard samples. Continue until *K* rounds of training are completed.
3. Construct a strong regressor. After all iterations are completed, the output values of the *K* Random Forest base learners are weighted and combined according to their respective weight coefficients to obtain the final prediction value of the strong learner.

## 3. Empirical Results

### 3.1. Variable Selection and Data Description

In order to systematically study the systemic risk contagion effect of China’s financial system under the impact of climate risk, referring to Ma et al. [52], this paper uses the China Climate Change News Index (hereinafter referred to as CCCNI) as a proxy indicator of climate risk. The index is constructed by using news articles published by nine Chinese newspapers (People’s Daily, Guangming Daily, Xinhua Daily Telegraph, China News Service, Science and Technology Daily, Science Times, China Energy News, China Environment News and Global Times) to construct the frequency of words for natural disasters, climate governance, energy transition, climate cooperation and climate communication, and provides a daily frequency index. The data comes from the website of ISETS Energy Finance Network (IEFN) (http://www.cnefn.com/data/download/climate-attention-database/ (accessed on 1 November 2025)). This paper divides China’s financial system into six primary sub-markets: money market, capital market, commodity market, foreign exchange market, gold market and real estate market, and determines the measurement indicators for each secondary sub-market. The division of the financial system and the construction of the indicator system are shown in Table 1. This paper uses the daily closing price of each market indicator to calculate the corresponding logarithmic rate of return to capture the price change trend. The data comes from the Wind database. To prevent logarithmic returns from failing to adequately capture the peaked, heavy-tailed, and skewed characteristics of tail risk in financial markets, we first selected the EGARCH-SGED model to perform volatility processing on the market data. The sample period is from January 2010 to February 2025.

To reflect the international macroeconomic situation and the external macroeconomic tail risks affecting China’s financial system, this paper selects the following six macroeconomic state variables: WTI crude oil spot price, SP 500 stock index, US dollar index (DXY), USD/EUR exchange rate (USD/EUR), London gold spot price (XAUUSD), and China’s 10-year government bond yield (CN10Y).

### 3.2. Systemic Risks in China’s Financial Markets Under the Impact of Climate Risks

Under the impact of climate risk, the total connectedness of systemic risk in China’s financial market shows significant dynamic fluctuations. High-level fluctuations occurred in several key periods from 2010 to early 2025. For details on the specific fluctuation trends and driving mechanisms of each period, please refer to Appendix A.

**Figure 2:**
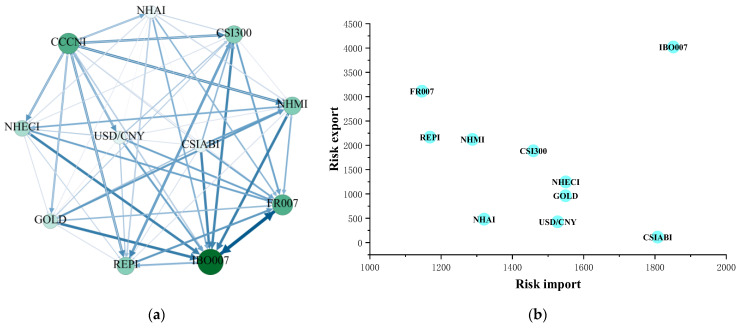
Systemic financial risk contagion in China under climate risk shock: (**a**) Contagion network; (**b**) Spillover and inflow of systemic financial risk.

This section focuses on the systemic risk spillover of China’s financial system under the impact of climate risk. A systemic risk contagion network of China’s financial system under climate risk shock is constructed based on the risk spillover matrix, as shown in Figure 2a. Representative indicators of climate risk and various financial sub-markets are used as nodes. The darkness and size of the node color represent the weighting degree, the edges between nodes represent the risk spillover between different markets, the arrows indicate the direction of risk spillover, and the thickness of the edges represents the magnitude of risk spillover (the same settings are used for the climate event analysis diagram below). Overall, there are relatively close risk correlations among financial markets. IBO007 (the interbank lending market) maintains strong connections with multiple markets and is located in the central region of the network, indicating its important role in risk transmission. To further identify key nodes in the risk transmission network of the financial system under climate risk shocks, this paper calculates the betweenness centrality (BC) and PageRank centrality (PR) of each node. The results show that FR007, CCCNI, and IBO007 (tied with NHMI and GOLD) have high BC values, indicating that these markets are located in many risk transmission paths and play a strong bridging role in cross-market risk transmission. CCCNI has a high betweenness centrality, indicating that climate risk is not only an important source of financial system risk but can also affect the risk transmission process between different markets through the financial network. Meanwhile, IBO007, FR007, and REPI have high PR values, indicating that the money market and the real estate market have high systemic importance in the entire risk network. Overall, climate risk nodes play a key connecting role in the risk transmission path, while monetary market-related nodes are important hubs for risk diffusion, reflecting the significant cross-market transmission characteristics of the financial system under the impact of climate risk.

Meanwhile, based on the risk spillover and inflow levels across the entire sample period, Figure 2b illustrates the systemic risk spillover and inflow into China’s financial system under the impact of climate risk. The scatter plot provides a visual analysis of the risk spillover and inflow in each market. Firstly, the bond market exhibits a high level of risk inflow and a low level of risk spillover. The repurchase market, on the other hand, shows a high level of risk spillover and a low level of risk spillover. The interbank lending market exhibits both strong risk inflow and spillover levels. The agricultural product market shows neither strong risk inflow nor spillover levels. From a market characteristic perspective, the bond market typically acts as a “reservoir” of funds, absorbing more risk from other markets. The repurchase market plays a crucial role in short-term financing; frequent interbank lending can easily make it a source of risk transmission, thus exhibiting high risk spillover and low inflow. The interbank lending market is an important venue for short-term fund adjustments among financial institutions; the intensity of fund flows makes both inflow and outflow of risk significant, resulting in strong risk inflow and spillover levels. The agricultural commodity market has a relatively low degree of direct connection with the financial market, and the interaction between funds and risks is relatively limited. Therefore, the levels of risk spillover and inflow are not strong.

By integrating network structure, centrality indicators, and risk spillover results, it can be observed that China’s financial system exhibits both direct risk transmission and indirect risk diffusion mechanisms under the impact of climate risk. From the perspective of direct connectivity, the money market is a crucial hub for risk transmission, with significant tail risk spillover relationships existing between different financial sub-markets. From the perspective of indirect connectivity, BC and PageRank indicators show that climate risk nodes, money market nodes, and the real estate market (REPI) are located in numerous risk propagation paths, further amplifying the risk diffusion effect through network structure. This result is consistent with theoretical expectations that climate risk will not be confined to a single market but will spread across markets through the internal connections within the financial system. This indicates that climate risk not only directly affects specific financial markets but also spreads to other markets through key nodes, generating systemic impacts. This provides an important basis for further analysis of the dynamic evolution characteristics of risk relationships and their transmission effects.

### 3.3. Climate Risks Spillover into China’s Financial System

**Figure 3:**
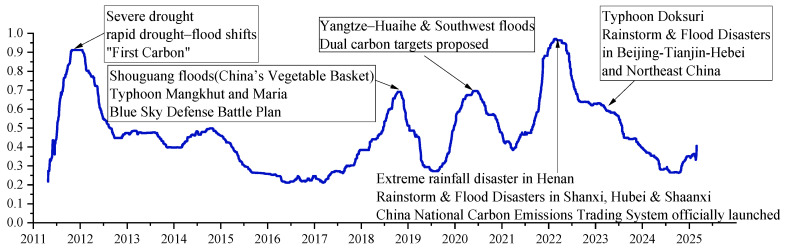
Climate risk spillover level into China’s financial system. Notes: “First Carbon” refers to the first time that China incorporated carbon intensity into its official assessment system with binding targets. Blue Sky Defense Battle Plan refers to the Three-Year Action Plan to Win the Blue Sky Defense Battle in China. Yangtze–Huaihe refers to the Yangtze and Huai River basins; Southwest China includes Sichuan, Chongqing, Shaanxi, Gansu, and Yunnan. Dual carbon target refers to China’s goals of carbon peaking and carbon neutrality.

**Figure A1:**
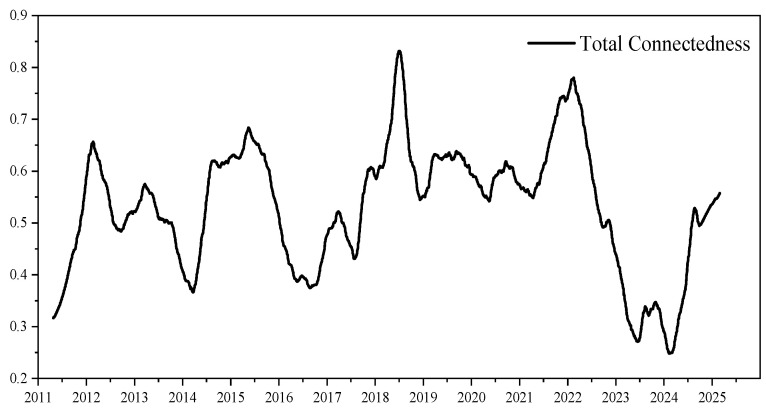
Overall Correlation of Systemic Risk in China’s Financial System under Climate Risk Shocks.

Figure 3 shows the changes in the level of climate risk spillover into China’s financial system over time. Compared with the total connectedness of climate risk impact on China’s financial system (see Figure A1 in Appendix A), it can be seen that, except for 2015 when the total connectedness was relatively high and dominated by extreme volatility in the domestic capital market, the overall risk spillover level of climate risk into China’s financial system was relatively high during the periods of high total connectedness at the end of 2011-beginning of 2012, 2018, and 2020–2021. Furthermore, the overall risk spillover level of climate risk into China’s financial system showed a significant rebound in 2024, which further confirms that climate risk has a significant impact on the level of systemic risk in China’s financial system.

**Figure 4:**
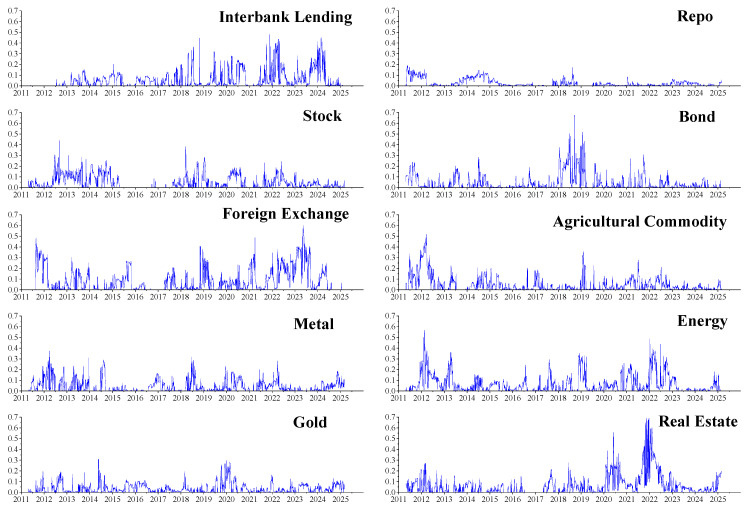
Risk spillover levels of climate risk to various financial sub-markets.

Figure 4 illustrates the changes in the risk spillover level of climate risk to various financial sub-markets over time. Combined with Figure 3, it can be seen that the overall risk spillover level of climate risk to the Chinese financial system was relatively high at the end of 2011-beginning of 2012, 2018, and 2020–2021, with most financial sub-markets experiencing significant risk spillover. The interbank lending market, represented by the IBO007 7-day interbank offered rate, primarily reflects the immediate liquidity situation within the banking system, rather than long-term risk. Its fluctuations are largely guided and regulated by the central bank’s open market operations. Therefore, the risk spillover of climate risk to the interbank lending market has a lag. Furthermore, in terms of overall spillover levels, the risk spillover level of climate risk to the repurchase market is relatively small, while the risk spillover level to the bond market and the real estate market is relatively large. The relatively small risk spillover level of climate risk to the repurchase market is mainly due to its short-term nature and collateral mechanism. Another market in the money market, the interbank lending market, relies heavily on the creditworthiness of counterparties due to its unsecured credit financing characteristics. This allows climate risk to have a more direct impact on financing costs by affecting bank asset quality and increasing credit risk. When banks’ expected solvency declines due to holding high-carbon assets or facing physical shocks, the risk premium they face in the unsecured lending market tends to rise more easily. Among various financial markets, the bond market and the real estate market are most affected by the spillover effects of climate risk. This is mainly because bond pricing depends on the stability of the issuer’s future cash flow and creditworthiness. Climate transition risks weaken the profitability and solvency of companies in high-carbon industries, increasing their default risk and triggering adjustments in bond credit spreads. Simultaneously, climate physical risks can indirectly impact the issuer’s operational continuity and debt repayment foundation by disrupting production facilities and supply chain integrity. The real estate market, due to the immobility and physical nature of its assets, is more susceptible to the impact of climate physical risks. For example, sea-level rise and extreme weather events can damage buildings and affect asset values through increased insurance costs and changes in regional economic attractiveness. Furthermore, both types of markets rely heavily on the discounted value of long-term, stable expected cash flows. Uncertainty arising from climate risk could affect their valuation basis and drive up risk premiums. Therefore, the bond and real estate markets, due to their long maturities, cash flow dependence, and physical vulnerability, may become important vehicles for the transmission and spread of climate risk within the financial system.

In summary, the impact of climate risk on China’s financial system exhibits a clear cross-market transmission characteristic. Climate risk not only increases the overall risk spillover level of the financial system but also has differentiated impacts on different financial sub-markets, with the bond market and real estate market experiencing relatively stronger risk spillovers. This result indicates that climate risk can propagate within the financial system through channels such as credit risk, asset pricing, and market expectations, altering the risk correlations between different markets, thus further revealing the main characteristics of the risk transmission path in China’s financial system under the impact of climate risk.

### 3.4. Systemic Risk Contagion in China’s Financial System Under Extreme Climate Events

#### 3.4.1. Systemic Risk Contagion Impact of Typhoon Doksuri on China’s Financial System

Typhoon Doksuri, the fifth typhoon of 2023, made landfall as a strong typhoon near Jinjiang, Fujian Province, at around 9:55 AM on 28 July. It was the strongest typhoon to make landfall in mainland China in 2023 and the second strongest to hit Fujian since 1949. Its impact covered five provinces: Fujian, Zhejiang, Anhui, Jiangxi, and Guangdong. In late July and early August, the remnants of Typhoon Doksuri caused extreme heavy rainfall in the Beijing-Tianjin-Hebei region, triggering severe flooding, landslides, and mudslides. In early August, influenced by the northward movement of the typhoon’s remnants and a westerly trough, many parts of Northeast China experienced heavy rainfall, leading to flooding. This disaster affected over 9.65 million people nationwide, requiring the emergency relocation of over 2.1 million people. Numerous houses were damaged, and over 1000 hectares of crops were affected, resulting in direct economic losses totaling 202.26 billion yuan. The scale and severity of the disaster were unprecedented. Considering the combined effects of the typhoon and its remnants, and given the time required for the recovery of production and daily life after the typhoon, this paper selects 28 July to 15 August 2023 as the “Doksuri” typhoon event, and selects an equal number of trading days before and after the event as the intervals before and after the event to study the risk network and total connectedness.

Data shows that the TC value surged from 46.6879 to 57.9593 from the “pre-event” window to the “during-event” window. This significant jump in TC during the event period confirms that extreme weather events, as a typical exogenous negative shock, can be rapidly transmitted and amplified within the financial network through both real economic and sentiment channels, leading to a short-term increase in total connectedness and directly increasing the vulnerability of the entire system. This finding aligns with the classic theory of financial risk contagion, demonstrating that climate factors have become an undeniable source of financial instability.

A more profound finding lies in the asymmetry and persistence of risk. The TC value in the ex-post window did not recover to the pre-ex-post level, but remained at a relatively high level of 51.2690. The impact of climate risk shocks is not fleeting. Even after the direct physical shock ends, its effects on the financial system persist, creating a lasting “scarring effect.” This structural change may stem from multiple factors: first, the asset quality damage caused by disasters is long-term; second, the post-disaster reconstruction process brings sustained credit pressure to the regional financial system; and third, market participants may therefore reassess the long-term premium of climate risk, thereby systematically raising the overall level of risk aversion.

**Figure 5:**
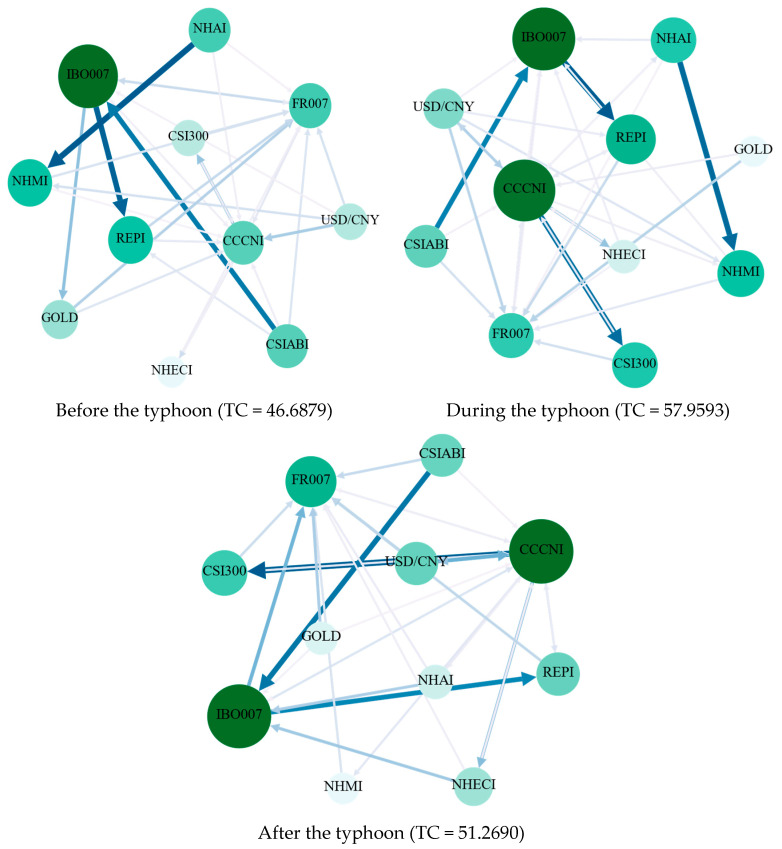
Network diagram of systemic risk contagion in China’s financial system before, during and after Typhoon Doksuri.

The network diagrams before, during, and after the typhoon event in Figure 5 show that the network connections were tighter during the event, and the role of nodes representing climate risk became more prominent after the event. Therefore, climate physical risk shocks not only trigger short-term contagion peaks in financial systemic risk but may also cause long-term vulnerability. While short-term post-disaster liquidity support policies can effectively smooth the peak of risk impulses, they are insufficient to address the sustained rise in risk levels. This warns us that financial stability policies should not only focus on post-disaster emergency hedging but also establish long-term mechanisms to enhance system resilience in response to the challenges posed by the normalization of climate risks. Therefore, the macroprudential policy framework must proactively incorporate climate risk factors and strive to enhance the overall resilience of the financial system. For example, long-term mechanisms such as conducting climate stress tests, promoting catastrophe insurance securitization, and guiding capital investment towards climate adaptation areas can be used to address the financial stability challenges under the normalization of climate risks.

Further examination of the centrality changes in key nodes, as shown in Table 2, reveals that CCCNI, IBO007, and FR007 consistently ranked among the top three in betweenness centrality across the pre-, mid-, and post-event periods. This indicates that climate risk and the money market remain crucial in the indirect transmission path of financial system risks. CCCNI consistently ranked first in betweenness centrality across all stages, demonstrating the significant bridging role of climate risk nodes in cross-market risk propagation. From a PageRank perspective, CCCNI rose from third before the event to first after, while CSI300 entered the top three, reflecting the increasing influence of climate risk in the risk network and its gradual diffusion to the stock market. This result answers the research question posed in this paper regarding “the pathways through which climate risk affects the financial system,” demonstrating that climate risk is not only a significant source of risk shocks but also leverages the financial network structure to drive the continuous diffusion of risk across different markets.

Overall, the impact of Typhoon Doksuri not only significantly enhanced the direct risk correlation within the financial system but also elevated the core position of climate risk nodes in the risk propagation network, revealing the dynamic evolution of climate physical risk from short-term external shocks to long-term systemic vulnerability.

#### 3.4.2. Contagion Impact of “Dual Carbon Targets” on Systemic Risk in China’s Financial System

In September 2020, China proposed that “carbon dioxide emissions should peak before 2030, and efforts should be made to achieve carbon neutrality before 2060.” On 16 July 2021, China’s national carbon emissions trading market was officially launched. This is the world’s largest carbon market, and its launch signifies that China, for the first time at the national level, has marketized the allocation of carbon emission rights as a scarce resource, marking the transition of China’s dual-carbon strategy from the goal declaration stage to the substantive implementation stage of “market mechanism + top-level design”. Therefore, this paper selects the period from the proposal of the dual-carbon goals in September 2020 to the official launch of the national carbon emissions trading market in July 2021 as representative dual-carbon events. The period before September 2020 is considered before the events, and the period after July 2021 is considered after the events. To avoid incomparability of total connectedness due to different time intervals, the average daily total connectedness (ATC) is used here.

**Figure 6:**
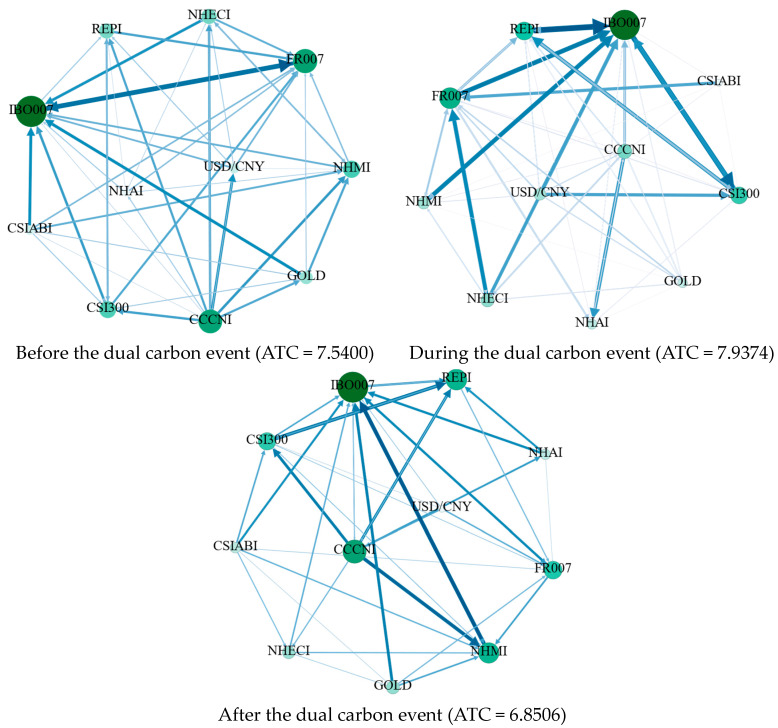
Network diagram of systemic risk contagion in China’s financial system before, during and after the National Carbon Emissions Market Event.

In Figure 6, before the “dual carbon” target was proposed, ATC = 7.5400. Fluctuations across industries mainly stemmed from conventional economic and financial factors. Climate transition risk had not yet become an independent driving factor widely priced in by the market and triggering systemic resonance. Between the formal proposal of the “dual carbon” target and the official launch of the national carbon emissions trading market, ATC significantly increased to 7.9374. Climate transition risk significantly impacted the market, spreading and resonating across industries, thus increasing the total connectedness of the entire economic system. After the event, ATC = 6.8506, falling from its peak and significantly below the pre-event benchmark level. This is because the carbon market, through the “polluter pays” principle, transforms abstract carbon emission constraints into clear, tradable carbon prices, providing a market-based pricing mechanism for transition risks.

Following the event, the ATC (6.8506) not only declined significantly compared to the period before the event but also fell below the pre-event level (7.5400), indicating a weakening of risk transmission within the financial system. This result is largely consistent with our expectations. Theoretically, the carbon market can promote the reallocation of climate risks through market-based pricing mechanisms, reducing the degree of risk accumulation that was originally concentrated within the financial system. It indicates that the implementation of the “dual carbon” policy and its tools optimized the market risk pricing mechanism. Transitional policies internalized previously unpriced “climate externalities”, thereby reducing the intrinsic total connectedness of the entire economic system. Changes in network structure further validate this conclusion. Looking at the three network diagrams before, during, and after the event, the risk exhibited a pattern of first increasing and then decreasing. Before the event, network nodes were sparsely connected, risk factors were dispersed and independent, systemic risk contagion paths were limited, and the overall risk level was low. During the event, node connections were dense and complex. Policy-induced uncertainty strengthened risk linkages, accelerated risk transmission, significantly increased the potential contagion of systemic risk, and the risk level rose sharply. After the event, node connections returned to a sparse and orderly state, the scope and intensity of core node connections decreased, the introduction of the carbon emission rights market mitigated the risk, transmission paths decreased, and the systemic risk level declined.

Further examination of the centrality changes at key nodes, as shown in Table 3, reveals that CCCNI, IBO007, and FR007 consistently ranked among the top three in betweenness centrality before and during the event, indicating that climate transition risks and the money market remain crucial pathways for the indirect transmission of financial system risks. After the official operation of the national carbon emission rights market, CCCNI dropped out of the top three in betweenness centrality, suggesting a weakening of the bridging role of climate transition risks in cross-market risk transmission. From a PageRank perspective, IBO007 and FR007 maintained high rankings before, during, and after the event, reflecting that the money market remains a significant influencing node in the risk network. This result answers the research question posed in this paper regarding “how climate transition risks affect the transmission of financial system risks,” demonstrating that in the initial stages of proposing “dual carbon” targets, policy uncertainty enhances market risk correlations, while as market mechanisms such as the carbon market gradually improve, the transmission effect of climate transition risks weakens, and the financial system risk network tends to stabilize.

It can be observed that climate risk can both increase the overall interconnectedness and risk transmission intensity of the financial system through physical risks such as typhoons, and alter the financial network structure and risk propagation paths through transitional risks such as “dual-carbon” policies. Whether physical or transitional, climate risk affects the risk transmission process of the financial system through key nodes and cross-market linkage mechanisms. These results further reveal the dynamic characteristics of the risk interconnectedness and transmission paths of China’s financial system under climate risk shocks, providing empirical evidence for understanding how climate risk evolves into systemic financial risk.

### 3.5. Machine Learning of Systemic Risk Early Warning Under Climate Risk Shocks

To further examine whether the indicators selected in the study on the contagion effect of systemic financial risk in China under climate risk shocks can effectively predict systemic risk, and to investigate whether the selected indicators can be included in the crisis early warning indicator system, machine learning methods are used to determine the rationality of the indicators selected in the previous section and to provide early warning of systemic financial crises. Based on understanding the contagion effect of systemic financial risk in China under climate risk shocks, this paper further constructs an early warning system for systemic financial risk in China under climate risk shocks. This system integrates network structure characteristics and macroeconomic influencing factors, selecting multi-dimensional indicators including CoVaR, betweenness centrality of CCCNI (BC-CCCNI), VIX index, China’s term spread (TermSpread), the US effective federal funds rate (EFFR) and the ChinaBond-China Green Bond Index (GreenBond), as well as various financial market measurement indicators and macroeconomic state variables, to comprehensively capture the total connectedness of the financial system.

#### 3.5.1. Model Selection and Algorithm Design

To address the common nonlinear relationships and complex interaction effects in financial risk data, this study employs a Random Forest Regression algorithm (RF-AdaBoost) based on an improved AdaBoost. For data partitioning, the complete dataset is randomly split in an 8:2 ratio, resulting in an 80% training set and a 20% test set. The training set is used for learning and optimizing model parameters, while the test set is used to evaluate the model’s generalization ability. This ensemble learning method combines the robustness of Random Forest with the adaptive enhancement capabilities of AdaBoost. By iteratively adjusting sample weights, it significantly improves the model’s accuracy in identifying complex risk patterns. Random forest itself effectively reduces the risk of overfitting by constructing multiple decision trees and integrating their predictions. The AdaBoost mechanism further optimizes the model’s focus on difficult samples, enabling the overall early warning system to maintain learning effectiveness on 80% of the training data while exhibiting stronger generalization ability on the 20% of the test data. From a methodological perspective, Random Forest (RF) exhibits strong resistance to overfitting and robustness, effectively handling high-dimensional data and complex nonlinear relationships. However, its ability to identify extreme risk states is relatively limited, and its model interpretability is weak. AdaBoost, on the other hand, enhances learning on difficult samples by adaptively adjusting sample weights, achieving high prediction accuracy. However, it may suffer from insufficient stability when sample fluctuations are large. To fully leverage the advantages of both methods, this paper constructs an RF-AdaBoost hybrid model to balance model stability and prediction accuracy. It should be noted that Random Forest, AdaBoost, and their hybrid models focus more on improving predictive ability than on identifying causal relationships and economic mechanisms between variables. Therefore, the machine learning analysis in this section primarily serves to verify the effectiveness of the risk warning system constructed above and to validate the predictive ability of relevant indicators for changes in systemic risk from a predictive perspective.

#### 3.5.2. Model Performance Evaluation

We conducted a comprehensive evaluation of the RF-AdaBoost model’s performance on both the training and test sets, and the results are shown in Table 4. The evaluation metrics cover multiple dimensions, including mean squared error (MSE), root mean squared error (RMSE), mean absolute error (MAE), mean absolute percentage error (MAPE), coefficient of determination (R^2^), and residual predictive deviation (RPD), ensuring a multi-faceted assessment of the model’s performance.

**Figure 7:**
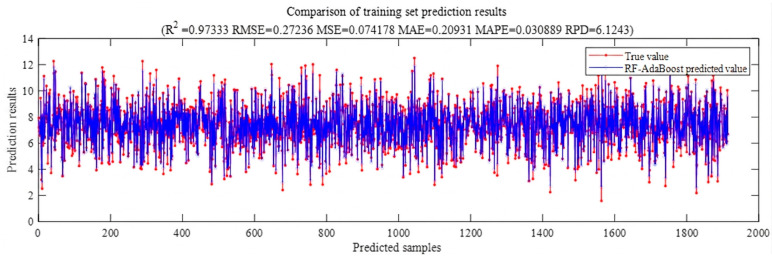
Comparison of prediction results for the training set.

As shown in Figure 7, the prediction results on the training set indicate that the RF-AdaBoost model fits the true values well. This is consistent with the high training accuracy (97.333%), suggesting that the model effectively captures the underlying patterns in the data. The error metrics on the training set are MSE = 0.0742, RMSE = 0.2724, MAE = 0.2093, and MAPE = 3.089%. These low error values, together with a high RPD of 6.1243, demonstrate the model’s excellent fitting performance and predictive accuracy.

**Figure 8:**
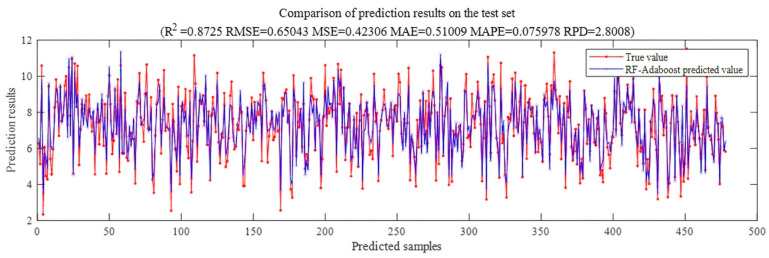
Comparison of prediction results on the test set.

As shown in Figure 8, the predicted values on the test set still exhibit a good agreement with the true values. Although the fit is slightly reduced compared to that of the training set, the model is still able to explain more than 85% of the variance in the test data (87.250%), indicating good generalization ability. The error metrics on the test set are MSE = 0.4231, RMSE = 0.6504, MAE = 0.5100, and MAPE = 7.598%. Although these values are higher than those of the training set, they remain within an acceptable range. In addition, the RPD value is 2.8008, which is greater than 2, further confirming that the model maintains good predictive performance on the test set.

**Figure 9:**
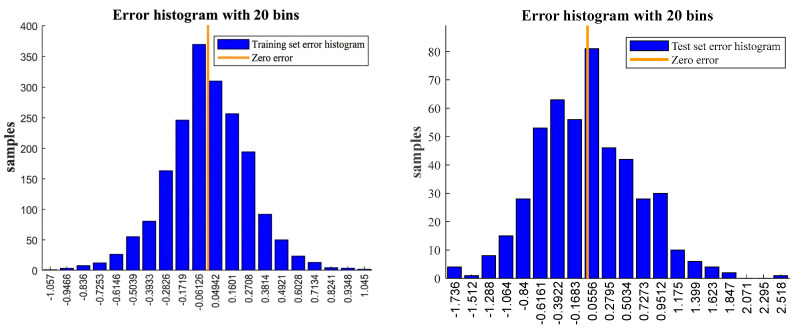
Error histograms of training and prediction sets.

To further analyze the model’s predictive performance, we plotted an error histogram with 20 bins in Figure 9. This histogram, based on the distribution of the model’s prediction residuals (the difference between the true and predicted values), clearly shows the frequency distribution characteristics of the errors. As can be seen from the histogram, the training set errors closely surround the zero value, exhibiting an approximately normal distribution, which is consistent with the relatively low MAE (0.5100) and RMSE (0.6504) metrics. While the test set error distribution shows a slight spread, it still maintains good overall symmetry, indicating that the model possesses good generalization ability.

This section focuses on the study of systemic financial risk contagion in China under the impact of climate risk. It constructs a financial systemic risk early warning system integrating network structure characteristics and macroeconomic factors, and the RF-AdaBoost algorithm employed demonstrates excellent performance. On the training set, the model accurately captures the patterns in the training data with low error and high fit. On the test set, it maintains good generalization ability, effectively explaining over 85% of the variance variation. The error histogram further validates that the error distribution on both the training and test sets reflects the model’s stability and reliability. This achievement provides effective tools and methodologies for timely and accurate early warning of systemic financial risk in China under the impact of climate risk, and for preventing the contagion and spread of risk. It also lays the foundation for further in-depth research into the interaction mechanism between climate risk and financial systemic risk, and for optimizing risk prevention and control strategies.

#### 3.5.3. Performance Comparison of Different Machine Learning Models in Risk Warning

To more comprehensively evaluate the performance advantages of the constructed early warning system and further clarify the applicability and superiority of the RF-AdaBoost algorithm in the early warning task of China’s financial systemic risk under the impact of climate risk, this study introduces Random Forest and AdaBoost as reference benchmarks, and conducts multi-model comparative analysis on the same dataset, based on the confirmation that the RF-AdaBoost single model has good predictive performance. The comparison results are shown in Table 5.

A horizontal comparison of the performance of RF, AdaBoost, and RF-AdaBoost models across three core dimensions—model fit, prediction accuracy, and model stability—shows that RF-AdaBoost demonstrates significant comprehensive advantages in early warning tasks for systemic financial risks in China under the impact of climate change.

In terms of goodness of fit, the RF-AdaBoost hybrid model achieved the best performance on the training set, with an R^2^ of 97.333%, outperforming RF (92.966%) and AdaBoost (69.678%). This suggests that the model is more capable of capturing complex patterns in the training data. On the test set, RF-AdaBoost also maintained its advantage, achieving an R^2^ of 87.250%, which is higher than that of RF and AdaBoost by 0.673% and 16.809%, respectively, indicating its superior generalization ability.

In terms of prediction accuracy, RF-AdaBoost’s MSE, RMSE, and MAE on the training set are 0.0742, 0.2724 and 0.20930, respectively, all lower than the other two models. On the test set, its MSE (0.4231), RMSE (0.6504), and MAE (0.5100) also remain at the lowest levels. Particularly noteworthy is that RF-AdaBoost’s MAPE is only 3.089% on the training set and 7.598% on the test set, meaning that the model’s relative prediction error is controlled within a low range, indicating good practical application value. In contrast, AdaBoost’s error metrics are significantly higher, with its test set MAPE reaching 12.061%, indicating relatively limited prediction accuracy.

From the perspective of model stability and reliability, the RPD metric provides an important reference. RF-AdaBoost achieves an RPD of 6.1243 on the training set, far exceeding the excellent model threshold (2.5), while its RPD on the test set is 2.8008, still within the excellent prediction capability range. RF’s test set RPD is 2.7323, indicating that its predictive ability was inferior to RF-AdaBoost. While Adaboost’s test set RPD is only 1.8393, reaching only a medium prediction capability level. This result indicates that RF-AdaBoost not only has high prediction accuracy but also better stability and reliability.

Comparative analysis of the performance degradation between the training and test sets allows for further assessment of the overfitting risk level of each model. RF’s R^2^ decreased by 6.389 percentage points from the training set to the test set, indicating some overfitting. AdaBoost model’s R^2^ on the test set is slightly higher than that on the training set, with a difference of only 0.763 percentage points. This phenomenon is usually related to the random sample partitioning and insufficient model fitting ability. RF-AdaBoost’s degradation was 10.083 percentage points. Although RF-AdaBoost exhibits a larger decline in R^2^ from the training set to the test set than RF, it still achieves the highest test-set R^2^ and the lowest prediction errors among all models. This suggests that the improvement in fitting ability does not come at the expense of substantial generalization loss.

In summary, RF-AdaBoost achieves complementary advantages by combining the mechanisms of RF and Adaboost. RF effectively controls model variance and improves stability. Adaboost enhances its learning ability on difficult samples by adaptively adjusting sample weights. In the task of early warning of systemic financial risks in China under the impact of climate risk, the RF-AdaBoost model significantly outperforms RF and AdaBoost in terms of fitting accuracy, generalization ability, and prediction stability. It is more suitable for handling complex interaction effects between multi-dimensional indicators, providing more reliable technical support for early warning of risk contagion.

#### 3.5.4. Importance Analysis of Variables in the RF-AdaBoost Model

To further identify the contribution of different variables to systemic risk early warning, this paper introduces the SHAP (Shapley Additive Explanations) method to analyze the importance of each variable in the early warning model, thereby identifying the marginal contribution of different variables to the systemic risk contagion early warning. Based on Shapley value theory, SHAP quantifies the impact of each input variable on the prediction results by calculating the marginal contribution of each variable to the model output under different subsets, and can capture nonlinearity and variable interaction effects.

**Figure 10:**
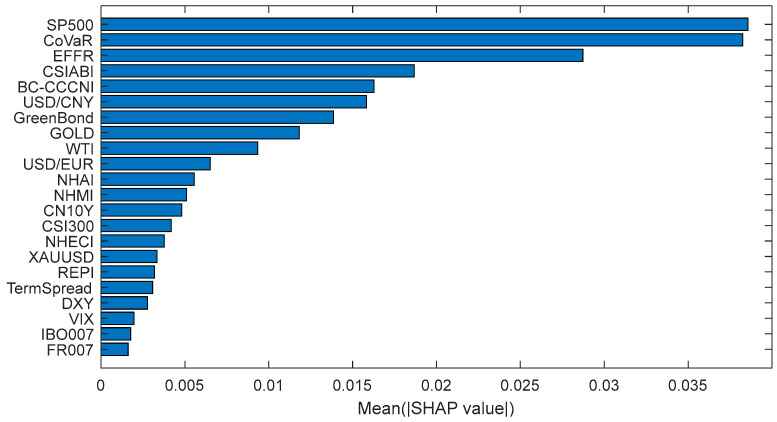
Feature importance ranking of the RF-AdaBoost model based on SHAP values.

Figure 10 shows the mean absolute SHAP values of each variable. The results indicate that variables such as SP500, CoVaR, EFFR, CSIABI, and BC-CCCNI are relatively important, suggesting that risk network characteristics and the macro-financial environment have strong explanatory power for early warning of systemic financial risk contagion. Notably, the climate risk news index betweenness centrality (BC-CCCNI) ranks among the top five most important variables, indicating that climate risk plays a significant role in the transmission of systemic financial risk. As a major external shock source driving changes in risk correlation structures, climate risk primarily affects the evolution of systemic risk by influencing the risk correlations and transmission paths between financial markets. The high importance of BC-CCCNI further validates the bridging role of climate risk in the financial network and demonstrates that incorporating climate risk network characteristics into the early warning model helps improve the ability to identify and warn of systemic risks.

These results validate the effectiveness of the systemic risk early warning system constructed in this paper under the background of climate risk, thus answering the second research question of this paper: “How to achieve effective early warning of systemic risk contagion?”

## 4. Conclusions

This paper, based on the China Climate Change News Index and combining EGARCH-SGED, LASSO-CoVaR, complex network analysis, and the RF-AdaBoost model, studies the systemic risk contagion in China’s financial system under the impact of climate risk shocks. The study finds that climate risk has become a significant exogenous shock source affecting the interconnectedness of China’s financial system. Significant cross-market risk transmission characteristics exist within the financial system. The interbank lending market and repurchase market play a strong pivotal role in the risk diffusion process. The bond market and real estate market exhibit strong risk absorption characteristics. Extreme climate events not only exacerbate short-term risk contagion but also have long-term impacts. The establishment of a national carbon market can help reduce the centrality of climate risk nodes in the financial network. Furthermore, the RF-AdaBoost model has good predictive accuracy and generalization ability in systemic risk early warning.

Based on the above research conclusions, this paper proposes the following policy recommendations. First, climate risk should be formally incorporated into the framework of financial stability monitoring and macro-prudential management. Research shows that climate risk strengthens inter-market risk correlations through the financial network and increases the overall vulnerability of the system. Therefore, regulators should focus on the dynamic changes in the relationship between climate risk and the financial market structure, incorporating climate risk indicators, tail risk spillover indicators, and network centrality indicators into a routine monitoring and stress testing system to improve the ability to identify the cross-market transmission of systemic risks.

Secondly, differentiated regulation should be implemented based on the risk transmission characteristics of different financial sub-markets. Empirical results show that the interbank lending market and the repurchase market have strong risk output characteristics and are important hubs for risk diffusion. The interbank lending market, with its unsecured credit financing characteristics, is more susceptible to climate risk shocks. Therefore, management of counterparty credit risk, liquidity risk, and concentration risk should be strengthened. The repurchase market should focus on preventing pro-cyclical deleveraging risks caused by fluctuations in collateral value. The bond market and the real estate market exhibit strong risk input characteristics and are more susceptible to cross-market risk shocks. Therefore, the bond market should strengthen credit risk management for high-carbon industries. The real estate market should strengthen regional climate risk assessment and collateral value monitoring to reduce the impact of extreme weather events on asset value and financing security.

Thirdly, the long-term impact effects of climate physical risks should be carefully prevented. Research has found that extreme weather events not only exacerbate the contagion of risks in financial markets in the short term, but may also create a lasting “scarring effect,” which can further spread through financial networks. Therefore, extreme weather scenarios should be incorporated into the stress testing system of financial institutions, disaster emergency liquidity support mechanisms should be improved, and the development of risk diversification tools such as catastrophe insurance and reinsurance should be promoted to enhance the financial system’s ability to mitigate climate physical risks.

Furthermore, the construction of a national carbon market should be further improved to leverage the market-based carbon pricing mechanism’s role in mitigating transition risks. Research results show that after the operation of the national carbon market, the centrality of climate risk nodes in the financial risk network has decreased, indicating that the carbon market can reduce the concentrated transmission of transition risks to some extent. Therefore, the carbon pricing mechanism should be further improved, the coverage of the carbon market expanded, and market pricing efficiency and the financial system’s ability to absorb climate transition risks enhanced.

Finally, the intelligent construction of a climate financial risk early warning system should be promoted. Research results show that the RF-AdaBoost model can effectively identify the dynamic changes in systemic risks under climate risk shocks. Therefore, risk network characteristics, climate risk indicators, financial market variables, and macroeconomic state variables can be incorporated into the early warning system. Machine learning methods can be used to improve the dynamic identification and real-time early warning capabilities for the contagion of high-dimensional, nonlinear systemic risks, thereby enhancing the foresight and accuracy of financial regulation.

## Figures and Tables

**Table 1:** Classification and Indicator System Construction of China’s Financial System.

	Primary Market	Secondary Market	Selected Indicators	Abbreviation
Financial System	Money Market	Interbank Lending Market	7-day Interbank Offered Rate	IBO007
		Repo Market	7-day Repo Fixing Rate	FR007
	Capital Market	Stock Market	CSI 300 Index	CSI300
		Bond Market	CSI Aggregate Bond Index	CSIABI
	Commodity Market	Agricultural Commodity Market	Nanhua Agricultural Index	NHAI
		Metal Market	Nanhua Metal Index	NHMI
		Energy Market	Nanhua Energy and Chemical Index	NHECI
	Foreign Exchange Market		USD/CNY Exchange Rate	USD/CNY
	Gold Market		Au9995 Gold Spot Price	GOLD
	Real Estate Market		CITIC Real Estate Industry Index	REPI

**Table 2:** Centrality Ranking of Key Network Nodes Before, During and After Typhoon Events.

Period	Betweenness Centrality Top3	PageRank Top3
Before the typhoon	CCCNI, IBO007, FR007	FR007, IBO007, CCCNI
During the typhoon	CCCNI, IBO007, FR007	FR007, CCCNI, IBO007
After the typhoon	CCCNI, FR007, IBO007	CCCNI, FR007, CSI300

**Table 3:** Centrality ranking of key network nodes before, during, and after the dual-carbon event.

Period	Betweenness Centrality Top3	PageRank Top3
Before the dual carbon event	CCCNI, FR007, IBO007/GOLD	IBO007, FR007, REPI
During the dual carbon event	CCCNI, IBO007, FR007	IBO007, FR007, CSI300
After the dual carbon event	IBO007, FR007, REPI	IBO007, FR007, NHMI

**Table 4:** Performance evaluation of the RF-AdaBoost model on the training and test sets.

Dataset	MSE	RMSE	MAE	MAPE	R^2^	RPD
training set	0.0742	0.2724	0.2093	3.089%	97.333%	6.1243
test set	0.4231	0.6504	0.5100	7.598%	87.250%	2.8008

**Table 5:** Comparison results of the three models.

Model	Dataset	MSE	RMSE	MAE	MAPE	R^2^	RPD
RF	training set	0.1956	0.4423	0.3417	5.029%	92.966%	3.7717
	test set	0.4453	0.6673	0.5242	7.809%	86.577%	2.7323
AdaBoost	training set	0.8586	0.9266	0.7406	10.876%	69.678%	1.8160
	test set	0.9201	0.9592	0.7889	12.061%	70.441%	1.8393
RF-AdaBoost	training set	0.0742	0.2724	0.2093	3.089%	97.333%	6.1243
	test set	0.4231	0.6504	0.5100	7.598%	87.250%	2.8008

## Data Availability

The original contributions presented in this study are included in the article. Further inquiries can be directed to the corresponding author.

## Abbreviations

The following abbreviations are used in this manuscript:

VaR	Value-at-Risk
CoVaR	Conditional Value-at-Risk
LASSO	Least Absolute Shrinkage and Selection Operator
RF	Random Forest
AdaBoost	Adaptive Boosting
RF-AdaBoost	AdaBoost-Enhanced Random Forest
GARCH	Generalized Autoregressive Conditional Heteroskedasticity
EGARCH	Exponential Generalized Autoregressive Conditional Heteroskedasticity
SGED	Skewed Generalized Error Distribution
ARIMA	Autoregressive Integrated Moving Average
TC	Total Connectedness
ATC	Average Daily Total Connectedness
RIS	Risk In-strength
ROS	Risk Out-strength
BC	Betweenness Centrality
PR	PageRank Centrality
CCCNI	China Climate Change News Index
IBO007	7-day Interbank Offered Rate
FR007	7-day Repo Fixing Rate
CSI300	CSI 300 Index
CSIABI	CSI Aggregate Bond Index
NHAI	Nanhua Agricultural Index
NHMI	Nanhua Metal Index
NHECI	Nanhua Energy and Chemical Index
USD/CNY	USD/CNY Exchange Rate
GOLD	Au9995 Gold Spot Price
REPI	CITIC Real Estate Industry Index
WTI	WTI Crude Oil Spot Price
SP500	SP 500 Stock Index
DXY	US Dollar Index
USD/EUR	USD/EUR Exchange Rate
XAUUSD	London Gold Spot Price
CN10Y	China’s 10-year Government Bond Yield
BC-CCCNI	Betweenness Centrality of CCCNI
VIX	VIX Index, the Cboe Volatility Index
TermSpread	China’s Term Spread (10-year minus 1-year government bond yields)
EFFR	US Effective Federal Funds Rate
GreenBond	ChinaBond-China Green Bond Index
MSE	Mean Squared Error
RMSE	Root Mean Squared Error
MAE	Mean Absolute Error
MAPE	Mean Absolute Percentage Error
R^2^	Coefficient of Determination
RPD	Residual Predictive Deviation
SHAP	Shapley Additive Explanations

## Appendix A

Figure A1 presents the overall dynamic correlation of China’s financial market under the impact of climate risk shocks from 2011 to early 2025 at the system level. It can be observed that the peak occurred in 2018, and the overall correlation level was relatively high in late 2011-early 2012, 2015, and 2020–2021.

The peak in 2018 was the most significant, a result of the combined pressures of financial tightening, trade conflicts, climate policies, and extreme weather. The “financial deleveraging” policy, aimed at shrinking the shadow banking system, also led to liquidity strain throughout the financial system. The US tariffs on Chinese goods and the escalating trade war severely threatened export-oriented economies, including China’s, causing widespread market panic and forcing assets across sectors to rise and fall in tandem. Simultaneously, climate risks entered the macroeconomic risk framework with unprecedented intensity, playing a crucial catalytic and amplifying role. The hottest summer on record resulted in 33 days of high-temperature warnings nationwide, especially the severe flooding in Shouguang, Shandong in August—affecting the nation’s vegetable supply and causing vegetable prices to surge by nearly 30% in a short period, significantly pushing up inflation expectations. Furthermore, Typhoon Maria in July and Typhoon Mangkhut in September caused significant economic losses and production disruptions. On the other hand, related environmental policies imposed production restrictions on energy-intensive and polluting industries, placing continuous financial and operational pressure on these enterprises. The combined impacts of extreme weather and transformation policies have further dampened liquidity expectations in the financial system, creating a multi-layered shock with deleveraging and the trade war, thus increasing systemic risks.

In late 2011 and early 2012, the European debt crisis spread from Greece to core countries such as Italy and Spain, triggering panic in global financial markets. Simultaneously, on the one hand, the 12th Five-Year Plan explicitly set binding targets of reducing energy consumption per unit of GDP by 16% and carbon dioxide emissions per unit of GDP by 17%, signifying that high-intensity environmental regulation had become the norm. For the first time, carbon emission intensity was incorporated into the assessment system, and low-carbon pilot demonstrations for the gradual establishment of a carbon emission trading market were proposed, allowing the market to begin pricing in the transformation risks faced by high-carbon industries. On the other hand, climate and physical risks continued to impact the domestic market. The severe drought in central and southwestern China at the beginning of the year severely affected agricultural production and hydropower generation. Particularly noteworthy was the “sudden shift from drought to flood” event in the middle and lower reaches of the Yangtze River, where a sudden downpour after a prolonged drought triggered severe flooding, not only driving up food prices but also exacerbating power shortages due to reduced hydropower output, disrupting industrial production. These extreme weather events, combined with the external shock of the European debt crisis, collectively increased the overall interconnectedness and risk transmission levels of the financial markets. In 2015, the overall interconnectedness of China’s financial markets peaked due to a systemic stock market crash triggered by a “thousand-stock limit down,” leading to a rapid depletion of liquidity through panic selling. Simultaneously, the People’s Bank of China announced improvements to the RMB exchange rate fixing mechanism on 11 August, causing a sharp short-term depreciation of the RMB. From 2020 to 2021, the overall interconnectedness of China’s financial markets remained high and gradually strengthened, primarily due to the shared macroeconomic shock of the COVID-19 pandemic. This led to indiscriminate selling of various assets, followed by a synchronized rebound due to global liquidity easing, ensuring that market volatility was consistently driven by the pandemic’s progress and policy responses. Meanwhile, the introduction of China’s “dual carbon” target in September 2020 triggered a market revaluation, with funds flowing from traditional energy sectors to green technology. This structural shock significantly increased market volatility and interconnectedness. Coupled with extreme weather events such as the devastating rainstorms in Henan, which impacted supply chains and caused localized inflation, the resonance intensity of market-wide repricing was further amplified. In 2022, as the impact of the pandemic faded and structural policies diverged, the market focus shifted from systemic resonance to sector differentiation, resulting in a significant decrease in overall correlation. In 2024, against the backdrop of a sluggish global economic recovery and persistent geopolitical risks, frequent extreme heat waves and floods, coupled with transformation policies, created dual pressures. These events significantly enhanced cross-industry risk transmission by impacting supply chains, driving up inflation expectations, and reshaping the energy landscape, leading to a marked rebound in the overall correlation of the financial markets.
